# Umbilical Cord Blood-Derived Exosomes From Very Preterm Infants With Bronchopulmonary Dysplasia Impaired Endothelial Angiogenesis: Roles of Exosomal MicroRNAs

**DOI:** 10.3389/fcell.2021.637248

**Published:** 2021-03-25

**Authors:** Xin-qi Zhong, Qin Yan, Zhuang-gui Chen, Chun-hong Jia, Xiu-hong Li, Zi-yan Liang, Jian Gu, Hui-ling Wei, Chang-yu Lian, Jing Zheng, Qi-liang Cui

**Affiliations:** ^1^Department of Neonatology, Third Affiliated Hospital of Guangzhou Medical University, Guangzhou, China; ^2^Key Laboratory for Major Obstetric Diseases of Guangdong Province, Guangzhou, China; ^3^Department of Gynecology, Shanghai First Maternity and Infant Hospital, Tongji University School of Medicine, Shanghai, China; ^4^Department of Pediatrics and Department of Allergy, The Third Affiliated Hospital, Sun Yat-sen University, Guangzhou, China; ^5^Department of Maternal and Child Health, School of Public Health, Sun Yat-sen University, Guangzhou, China; ^6^Department of Obstetrics and Gynecology, University of Wisconsin–Madison, Madison, WI, United States

**Keywords:** bronchopulmonary dysplasia, microRNA, angiogenesis, exosome, preterm infants

## Abstract

Premature infants have a high risk of bronchopulmonary dysplasia (BPD), which is characterized by abnormal development of alveoli and pulmonary vessels. Exosomes and exosomal miRNAs (EXO-miRNAs) from bronchoalveolar lavage fluid are involved in the development of BPD and might serve as predictive biomarkers for BPD. However, the roles of exosomes and EXO-miRNAs from umbilical cord blood of BPD infants in regulating angiogenesis are yet to be elucidated. In this study, we showed that umbilical cord blood-derived exosomes from BPD infants impaired angiogenesis *in vitro.* Next-generation sequencing of EXO-miRNAs from preterm infants without (NBPD group) or with BPD (BPD group) uncovered a total of 418 differentially expressed (DE) EXO-miRNAs. These DE EXO-miRNAs were primarily enriched in cellular function-associated pathways including the PI3K/Akt and angiogenesis-related signaling pathways. Among those EXO-miRNAs which are associated with PI3K/Akt and angiogenesis-related signaling pathways, BPD reduced the expression of hsa-miR-103a-3p and hsa-miR-185-5p exhibiting the most significant reduction (14.3% and 23.1% of NBPD group, respectively); BPD increased hsa-miR-200a-3p expression by 2.64 folds of the NBPD group. Furthermore, overexpression of hsa-miR-103a-3p and hsa-miR-185-5p in normal human umbilical vein endothelial cells (HUVECs) significantly enhanced endothelial cell proliferation, tube formation, and cell migration, whereas overexpressing hsa-miR-200a-3p inhibited these cellular responses. This study demonstrates that exosomes derived from umbilical cord blood of BPD infants impair angiogenesis, possibly via DE EXO-miRNAs, which might contribute to the development of BPD.

## Introduction

Very preterm infants (VPI, gestational age <32 weeks) account for 10% of preterm birth infants, and its prevalence keeps increasing in the world (Blencowe et al., 2013). Bronchopulmonary dysplasia (BPD) is one of the most severe respiratory complications in VPI, leading to fetal developmental delay and mortality (Reiterer et al., 2019). Development of BPD is attributed to a wide range of factors including use of ventilator, hyperoxia therapy, and other perinatal risk factors (Jobe and Bancalari, 2001). However, pathogenesis and the underlying mechanisms of BPD remain elusive.

The main pathological features of BPD include developmental retardation of alveoli and microvascular dysfunction (Kalikkot Thekkeveedu et al., 2017). The BPD-impaired pulmonary microvascular endothelium is primarily induced by hyperoxia (Buczynski et al., 2013). The decreased expression of pro-angiogenesis factors, such as vascular endothelial growth factor (VEGF) in BPD patients, is associated with pulmonary vasculature dysfunction (Bhatt et al., 2001; Alvira, 2016). In neonatal rats, BPD also impairs function of lung endothelial progenitor cells which are essential for lung microvascular growth and development (Alphonse et al., 2014). In addition, the number of endothelial progenitor cells decreased in the umbilical cord blood of the preterm infants with subsequent development of BPD (Bertagnolli et al., 2017). It is well-known that suppression of angiogenesis restrains the alveolarization during lung development in rats (Jakkula et al., 2000). On the other hand, improvement of pulmonary angiogenesis is beneficial for the alveolar development, which might prevent the development of BPD in animal models (Stenmark and Balasubramaniam, 2005). Thus, identifying the intrinsic factors which induce angiogenesis impairment will advance our understanding of BPD development and help us develop novel therapies and predictors for BPD.

Exosomes are nanosized extracellular vesicles with sizes ranging from 30 to 120 nm in diameter. Exosomes express a set of markers, such as cluster of differentiation 63 (CD63), Alix/apoptosis-linked gene-2 (ALG-2)-interacting protein X (Alix), and tumor susceptibility gene 101 protein (TSG101) (Willms et al., 2016; Salomon and Rice, 2017). Exosomes contain a number of signaling molecules including microRNAs (miRNAs), proteins, and messenger RNAs, which can be delivered as signaling molecules between cells. The function of exosomes varies in different cell types (Ghaemmaghami et al., 2020; Hashemian et al., 2020; Sadri Nahand et al., 2020; Amiri et al., 2021). Specially, exosomes derived from endothelial progenitor cells can promote the angiogenesis of pulmonary microvascular endothelial cells (Zhang et al., 2019). Exosomes derived from mesenchymal stromal cells (MSC) strengthen the tube formation of human umbilical vein endothelial cells (HUVECs) and promote the vascularization and alveolarization in BPD rats (Braun et al., 2018). It has been reported that maternal living environment, hereditary background, and perinatal circumstances can significantly alter the composition of exosomes from umbilical cord blood (Lura et al., 2018). Till now, it remains unclear whether exosomes in umbilical cord blood affect fetal pulmonary angiogenesis, contributing to the development of BPD.

MicroRNAs (miRNAs) are one of the main components of exosomes which have been implicated in numerous disorders including BPD (Zhang et al., 2015; Lal et al., 2018). Thus, exosome-derived miRNAs are potential candidates as the therapeutic targets and the biomarkers and prognosis markers for BPD (Mianehsaz et al., 2019; Asgarpour et al., 2020; Nahand et al., 2020a, b). Recent evidence has shown that maternal and umbilical exosomes contain an array of miRNAs that critically regulate angiogenesis (Jia et al., 2018). Exosomes isolated from bronchoalveolar lavage fluid of BPD mice can reduce the expression of miR-876-3p, suggesting that exosomal miR-876-3p could serve as a miRNA biomarker of severe BPD and may be used as a target for treating BPD (Lal et al., 2018). However, it remains unknown if exosomal miRNA profiles of umbilical cord blood differ between the BPD-susceptible (BPD group) and BPD-resistant (NBPD group) very preterm infants and if these differences contribute to impaired angiogenesis in BPD. In contrast to the much invasive and harmful approach of obtaining neonatal bronchoalveolar lavage fluid, collection of umbilical cord blood is non-invasive and safer for preterm infants.

In this study, we hypothesized that the differential expression of umbilical cord blood-deprived EXO-miRNAs between the BPD group and NBPD group is a major contributing factor of abnormal angiogenesis in BPD. Angiogenesis is a complex process in which endothelial cell proliferation and migration are essential steps (Folkman and Shing, 1992). Several well-known pro-angiogenic factors, such as basic fibroblast growth factors (bFGF) and vascular endothelial growth factor (VEGF), and platelet-derived endothelial cell growth factor (PF-ECGF), stimulate angiogenesis through promoting endothelial cell proliferation and migration (Klagsbrun and D’Amore, 1991). Endothelial cell proliferation and migration are the most common indicators for *in vitro* angiogenesis (Nowak-Sliwinska et al., 2018). Thus, this study used endothelial cell proliferation and migration assays as well as tube formation *in vitro* to evaluate angiogenesis. We determined the effects of umbilical cord blood-derived exosomes collected from the BPD and NBPD of very preterm infants on angiogenesis. Differentially expressed (DE) EXO-miRNA profiles between these two groups were analyzed. We also determined the effects of the DE EXO-miRNAs on endothelial angiogenesis. We identified a set of DE EXO-miRNAs in umbilical cord blood-derived exosomes from BPD and NBPD infants and provided new insights for the developing diagnosis and therapeutic strategies for BPD.

## Materials and Methods

### Study Subjects and Biospecimen Collection

Patients with very preterm delivery (28–31^+6^ weeks) were recruited at the Department of Neonatology, the Third Affiliated Hospital of Guangzhou Medical University, China. Patient recruitments and blood sample collection were approved by the Ethics Review Board of the Third Affiliated Hospital of Guangzhou Medical University. All experiments were performed in accordance with the ethical standards as laid down in the 1964 Helsinki Declaration and its later amendments or comparable ethical standards.

The inclusion criteria of the subjects are as follows: pregnant women without complications such as preeclampsia, gestational diabetes mellitus, premature rupture of membranes, and vaginal bleeding, and without adverse pregnancy history and multiple pregnancy or infectious diseases including vaginitis, gingivitis, respiratory tract infection, and urinary tract infection. Participants and corresponding infants were divided into BPD group or NBPD group depending on whether the infants developed BPD or not.

Umbilical cord vein blood (∼20 ml) was collected immediately after parturition and kept at 4°C for 30 min, followed by centrifugation (3000 rpm at 4°C for 15 min) to obtain serum samples. The serum was aliquoted and stored at −80°C until further analysis.

### Exosome Isolation

The serum sample (2 ml/infant) was centrifuged at 16,000 *g* at 4°C for 20 min to remove any remaining cells and cell debris. The supernatant was filtered through a 0.22-μm filter and diluted with 1 ml sterile PBS. Next, polyethylene glycol 6000 (PEG6000, Sigma-Aldrich, United States) was added to the supernatant mixture (1:4). Afterward, the mixture was gently vortexed and kept in 4°C for 60 min, followed by centrifuging at 10,000 *g* for 20 min. The exosome pellet was resuspended in 200–500 μl sterile PBS (pH = 7.4) and stored it at −80°C.

### Exosome Identification

Exosome suspension (5 μl/infant) was diluted into a total volume of 10 μl, fixed with 2% paraformaldehyde (PFA), and stained with 2% uranyl acetate solution for 1 min, followed by air drying. The morphology of exosome was observed by a transmission electron microscope (JEOL-JEM1400, JEOL Ltd., United States) at an acceleration voltage of 80 kV. The size distribution of exosomes was determined by nanoparticle tracking analysis (NTA). The sample (5 μl) was diluted step by step with PBS (5 μl). Then, the NTA measurements were performed using a Particle Metrix ZetaView instrument (Particle Metrix GmbH, Germany) with laser and video camera module (Particle Metrix GmbH, Germany). Flow mode with tracking of the Brownian motion of nanoparticles was used. The expression of exosomal surface marker proteins (TSG101 and Alix) was determined by Western blotting.

### Cell Culture and Exosome Treatments

Human umbilical vein endothelial cells were obtained from Cellcook (Guangzhou, China). HUVECs were cultured and passaged in high-glucose Dulbecco’s Modified Eagle Medium (DMEM, Thermo Fisher Scientific, United States) supplemented with 10% fetal bovine serum (FBS, Gibco, United States). Endothelial cells were starved for 16 h and then treated with exosomes (50 μg/ml) or an equivalent vehicle for various times before performing cell proliferation, migration, and tube formation assays.

### MiRNA Transfection

MicroRNA transfection was conducted as described (Zhou et al., 2017). Hsa-miR-103a-3p mimic, hsa-miR-185-5p mimic, hsa-miR-200a-3p mimic, and miRNA mimic negative control were purchased from Sangon Biotech (Shanghai, China). The sequences of miRNA mimics are listed in Table 1. HUVECs were transfected with miRNA mimics (200 pmol) and equivalent mimic negative controls using Lipofectamine 2000 reagent (Thermo Fisher Scientific, United States) according to the manufacturer’s instructions. Overexpression of miRNAs was confirmed by qRT-PCR after 24 h of miRNA mimic transfection. Effects of these miRNA mimics on angiogenesis were assessed after 48 h of miRNA mimic transfection.

**TABLE 1:** MiRNA mimic sequences.

**miRNA mimics**	**Sequences**
hsa-miR-103a-3p	AGCAGCAUUGUACAGGGCUAUGA
hsa-miR-185-5p	UGGAGAGAAAGGCAGUUCCUGA
hsa-miR-200a-3p	UAACACUGUCUGGUAACGAUGU

### Western Blotting

Human umbilical vein endothelial cells were lysed in RIPA buffer (50 mM Tris-base, 150 mM NaCl, 0.1% SDS, 0.5% sodium deoxycholate, 1% Triton X-100, pH 6.7), followed by centrifugation to obtain total protein samples. The protein concentration in the sample was measured using BCA assay kit (Thermo Fisher Scientific, United States). Protein samples were electrophoresed in sodium dodecyl sulfate-polyacrylamide (SDS-PAGE) gel and transferred to polyvinylidene fluoride membranes (PVDF, Millipore, Billerica, United States). Blots were blocked with 5% skim milk in Tris-buffered saline containing 0.1% Tween-20 (TBST) for 1 h at room temperature. Then, the membranes were incubated with primary antibodies (anti-TSG101: ab125011, Abcam, 1:5000; anti-Alix: #2171, CST, 1:1000) at 4°C overnight, followed by incubation with the horseradish peroxidase-conjugated secondary antibodies (horseradish peroxidase-conjugated anti-mouse: ab205719, Abcam, 1:1000; horseradish peroxidase-conjugated anti-rabbit: ab6721, Abcam, 1:1000) at 37°C for 1 h. The blots were developed using Chemiluminescent ECL reagent (Forevergen, China), and their gray values were analyzed using Image J software (NIH, United States).

### Cell Proliferation Assay

Cells (1 × 10^6^ cells/ml) were seeded into 96-well plates and subjected to the indicated treatments. When the treatment period was over, CCK-8 (10 μl, Beyotime Company, China) was added into each well and the plate was further incubated for additional 4 h. Afterward, optical density (OD) at 490 nm was read by microplate reader (Shanghai Flash Spectrum Biological Technology Company, China).

### Tube Formation Assay

Forty-eight-well plates were coated with Matrigel (BD Biosciences, United States) according to the manufacturer’s instructions. HUVEC cells (1.6 × 10^4^ per well) were seeded on Matrigel-coated plates and treated with exosomes or equivalent vehicle. For the miRNA overexpression experiments, HUVECs (1.6 × 10^4^ per well) transfected with corresponding miRNA mimic were seeded on Matrigel-coated plates. Cells were then incubated at 37°C with 5% CO_2_. Three fields were captured in microscope (Olympus BX51, Japan) randomly at 6 h after treatments, and the tube formation was analyzed using the Image J software (NIH, United States).

### Cell Migration Assay

Cell migration assay was performed using transwell (8.0 μm pore size, Corning, NY, United States) according to the manufacturer’s instructions. HUVECs were adjusted to the cell density of 1 × 10^6^ cells/ml. Then, 100 μl cell suspension was added to the upper chambers and 600 μl complete medium was added to the lower chambers. Cells in the upper chambers were treated with exosomes or equivalent vehicle for 16 h. Cell seeding procedures were the same as above to study the migration of HUVECs transfected with miRNA mimic, but there was no exosome treatment. Migrated cells were fixed with 4% paraformaldehyde and stained with 1% crystal violet. Cell migration images were taken using a Nikon Eclipse Ti microscope (Tokyo, Japan). The number of migrated cells was counted using Image J software (NIH, United States).

### Next-Generation Sequencing (NGS) and Bioinformatics Analysis

Total RNAs of exosomes were obtained using MiniBEST Universal RNA Extraction Kit (Takara, Japan). The concentration and quality of total RNA was determined by NanoDrop ND1000 (Thermo Fisher Scientific, United States). Reverse transcription reaction and gene library preparation were performed using NEBNext^®^ Multiplex Small RNA Library (E7300L, NEB, United States) according to the manufacturer’s instructions, followed by assessment of the final library product using the Agilent Bioanalyzer 2100 system (Agilent, United States). The library was then sequenced in the Illumina HiSeq 4000 platform using the 150 bp paired-end sequencing strategy. Cluster 3.0 software (United States) was used to generate a heat map of DE miRNA between the two groups (FDR ≤ 0.001 and | Log2Ratio| ≥ 1). Gene ontology (GO) enrichment was analyzed using Gene Ontology Enrichment Analysis Software Toolkit (GOEAST) with default parameters, including the molecular functions, biological processes, and cellular components. KEGG Orthology Based Annotation System (KOBAS) software was used to analyze the Kyoto Encyclopedia of Genes and Genomes (KEGG) signaling pathways for the differential expressions of miRNAs.

### qRT-PCR Analysis

EXO-miRNAs were purified using a SeraMir Exosome RNA Purification Kit (System Biosciences, Mountain View, CA, United States), followed by miRNA cDNA generation using the TaqMan microRNA assay kit (Applied Biosystems, Foster City, CA, United States) according to the manufacturer’s instructions. Total RNAs of cultured cells were extracted using Trizol Reagent (Invitrogen, United States). Revert Aid first-strand cDNA synthesis kit (Fermentas, Life Sciences, Canada) was then used to generate cDNA. qRT-PCR analysis was performed using SYBR Premix Ex Taq^TM^ II in the ABI PRISM^®^ 7900HT System (Takara Biotechnology, Japan). Relative standard curve method (2^–ΔΔCT^) was used to determine the relative mRNA expression. The miRNA-specific forward primers were synthesized in Sangon Biotech (Shanghai, China) and the universal reverse primer provided by the TaqMan microRNA assay kit. U6 small nuclear RNA was used for normalization. The sequences of miRNA PCR primers were indicated in Table 2.

**TABLE 2:** Primer sequence for miRNA qPCR.

**Primers**	**Sequences**
hsa-miR-103a-3p-RT	GTCGTATCCAGTGCAGGGTCCGAGGTATT CGCACTGGATACGACTCATAG
hsa-miR-103a-3p-F	GCGAGCAGCATTGTACAGGG
hsa-miR-17-5p-RT	GTCGTATCCAGTGCAGGGTCCGAGGTATTCGC ACTGGATACGACCTACCT
hsa-miR-17-5p-F	GCGCAAAGTGCTTACAGTGC
hsa-miR-185-5p-RT	GTCGTATCCAGTGCAGGGTCCGAGGTATTCGCA CTGGATACGACTCAGGA
hsa-miR-185-5p-F	GCGTGGAGAGAAAGGCAGT
hsa-miR-200a-3p-RT	GTCGTATCCAGTGCAGGGTCCGAGGTATTCGCA CTGGATACGACACATCG
hsa-miR-200a-3p-F	GCGTAACACTGTCTGGTAA
hsa-miR-20b-5p-RT	GTCGTATCCAGTGCAGGGTCCGAGGTATTCGCAC TGGATACGACCTACCT
hsa-miR-20b-5p-F	GCGCAAAGTGCTCATAGTGC
hsa-miR-765-RT	GTCGTATCCAGTGCAGGGTCCGAGGTATTC GCACTGGATACGACCATCAC
hsa-miR-765-F	GCGTGGAGGAGAAGGAAG
Universe-R	GTGCAGGGTCCGAGGT

### Potential Target Gene Evaluation

The target genes of hsa-miR-185-5p were predicted using mirtabase^1^. qRT-PCR was used to verify cyclin-dependent kinase 6 (CDK6) and DNA methyltransferase 1 (DNMT1) in HUVECs after 24 h of overexpression of hsa-miR-185-5p. A previous study showed that miR-185-5p suppressed the expression of vascular endothelial growth factor A (VEGFA) in human ovarian microvascular endothelial cells (Matthay and Abman, 2018). Thus, we also tested if VEGFA was the target gene of hsa-miR-185-5p. The primer sequences of these target genes are shown in Supplementary Table 1.

### Statistical Analysis

Quantitative data were presented as the Mean ± SD. The unpaired Student’s *t*-test was carried out to analyze the differences between two groups. One-way ANOVA followed by Bonferroni’s *post hoc* test was used to compare the statistically significant differences between the means of three or more groups. Qualitative data in basic demographic information were compared using the Chi-square test. All statistical analyses were performed using SPSS 22 software. *P* < 0.05 indicates a statistically significant difference.

## Results

### Participants’ Clinical Characteristics

Demographic characteristics of maternal and preterm infants in BPD and NBPD groups are shown in Table 3. No significant differences were found in maternal age, sex ratio, incident rate of gestational diabetes, and Apgar score between BPD and NBPD infants. The gestational age of the BPD group was significantly smaller (*P* < 0.05) than that of the NBPD group, while the maternal body mass index (BMI) of the BPD group were significantly (*P* < 0.05) higher than that of the NBPD group. The birth weight, birth length, and head circumference of infants in the BPD group significantly (*P* < 0.05) decreased when compared with the NBPD group.

**TABLE 3:** The demographic characteristics of patients.

**Variables**	**NBPD**	**BPD**	***P* value**
Study population (N)	14	12	
Maternal age (years)	30.5 ± 3.2	29.6 ± 2.9	0.453
Gestational age (weeks)	31.4 ± 2.3	29.9 ± 1.0	0.032
Maternal BMI	20.5 ± 2.8	23.3 ± 3.9	0.047
Sex ratio (M/F)	10/4	4/8	0.052
Birth weight (kg)	1.7 ± 0.6	1.1 ± 0.3	0.002
Birth length (cm)	41.9 ± 5.7	37.4 ± 2.6	0.021
Head circumference (cm)	29.1 ± 2.5	26.7 ± 1.8	0.011
Apgar score	9.7 ± 0.5	9.3 ± 0.9	0.200

### Exosome Characteristics of NBPD and BPD Groups

**FIGURE 1:**
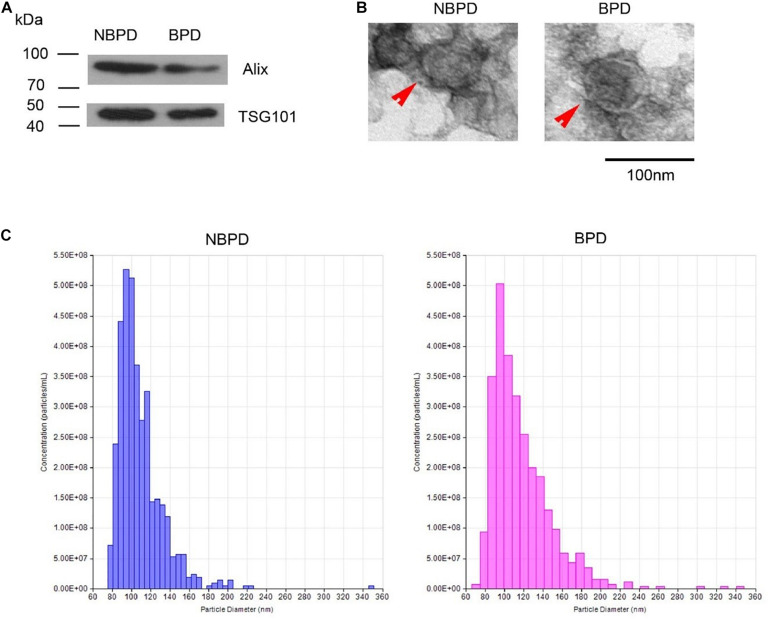
Exosome identification. **(A)** Expression of exosomal markers was determined by Western blotting. **(B)** Representative images showing morphology of exosome (red arrow) under transmission electron microscopy. **(C)** Nanoparticle size distribution measured by NTA. *n* = 3.

Exosome-surface markers, Alix and CD63, were confirmed in the vesicles of both groups (Figure 1A). The vesicles had an oval- or round-shaped appearance with a vesicle-like structure, as well as having a deep-stained lipid bilayer membrane in TEM images (Figure 1B). The diameter of the vesicles of both groups ranged from 30 to 150 nm, and the size distributions were comparable in both groups (Figure 1C). The results indicated that the isolated nanoparticles were exosomes, and they were comparable between two groups in term of the morphology, size, and size distribution.

### Exosomes of the BPD Group Impaired Endothelial Cell Proliferation, Migration, and Tube Formation

**FIGURE 2:**
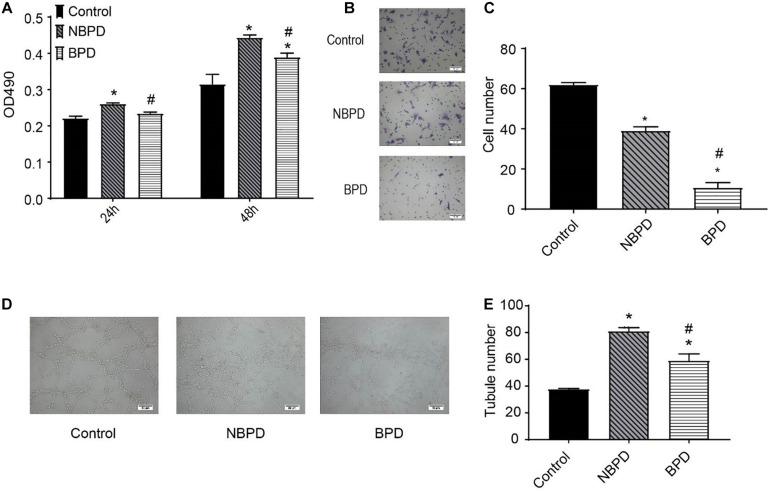
Effects of umbilical cord blood-derived exosomes on endothelial proliferation, migration, and tube formation. **(A)** HUVECs were treated with exosomes (50 μg/ml) from NBPD and BPD infants or vehicle for 24 h and 48 h, respectively. Cell proliferation was determined using CCK-8. **(B,C)** HUVECs were treated as indicated for 16 h, and number of migrated cells was determined by transwell. *^,#^*P* < 0.05 versus control and NBPD group, respectively. *N* = 3. Scale bar = 30 μm. **(D,E)** HUVECs were treated with exosomes or vehicle as indicated. Tube formation was determined at 6 h after treatments (scale bar = 50 μm). *, ^#^ *P* < 0.05 versus control and NBPD, respectively. *n* = 3.

We determined the effects of exosomes from NBPD and BPD groups on endothelial cell proliferation, migration, and tube formation (Figure 2). As shown in Figure 2A, exosomes from the NBPD group significantly (*P* < 0.05) increased cell proliferation when compared with the vehicle control group using an equivalent volume of PBS, whereas exosomes from the BPD group significantly (*P* < 0.05) inhibited cell proliferation when compared with the NBPD group at 24 h and 48 h. Exosomes from both NBPD and BPD groups inhibited (*P* < 0.05) endothelial migration when compared with vehicle control, and the inhibitory effect was further enhanced in the BPD vs. NBPD groups (Figures 2B,C). We also found that exosomes from the BPD group significantly (*P* < 0.05) reduced tube formation when compared with NBPD group (Figures 2D,E).

### Differential Expression of miRNA in Exosomes From NBPD and BPD Groups

**FIGURE 3:**
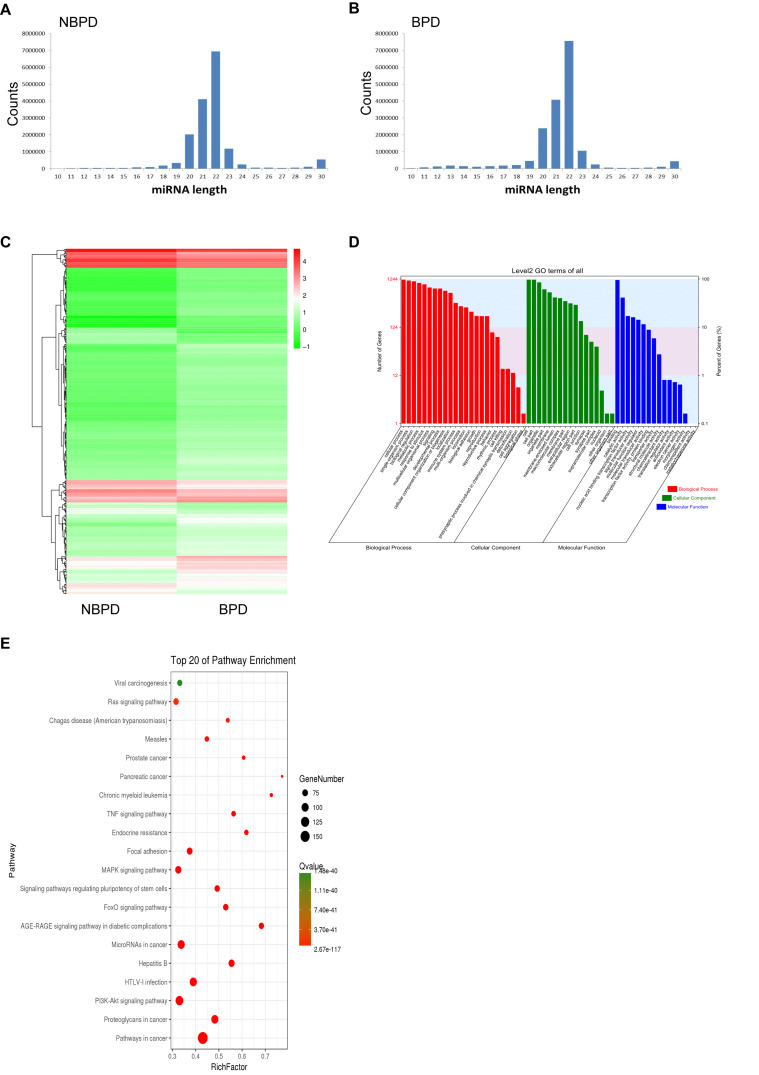
EXO-miRNA profile of umbilical cord blood from NBPD and BPD infants and the bioinformatics analysis of DE miRNAs. **(A,B)** The length distribution of EXO-miRNAs in NBPD and BPD patients. **(C)** Heat map of DE EXO-miRNAs between NBPD and BPD patients. **(D)** GO analysis results showing the functional categorization of DE miRNAs between NBPD and BPD infants. **(E)** KEGG pathway analysis showing the top 20 enriched KEGG pathways for DE EXO-miRNAs. *n* = 4.

We carried out NGS and bioinformatic analysis to determine the differential expression of EXO-miRNAs between NBPD and BPD groups. We obtained 20,912,199 and 20,435,555 clean reads in the NBPD and BPD groups, respectively, indicating that data had a desirable quality (Supplementary Table 2). As shown in Figures 3A,B, the length distribution of EXO-miRNAs in both NBPD and BPD groups dominantly ranged from 19 to 24 nucleotides (nt) with a peak at 22 nt, and it was comparable between two groups. A total of 2588 EXO-miRNAs were identified from both groups (Supplementary Table 3). A total of 418 DE EXO-miRNAs were found (Figure 3C and Supplementary Table 4), among which 328 miRNAs were upregulated while 90 miRNAs were downregulated. GO and KEGG analysis were performed on predicted target genes of DE EXO-miRNAs to exhibit the functional annotation. As shown in Figure 3D, most of the target genes were involved in biological processes, cellular component, and molecular function. Regarding the terms of the biological process, the most dominant categories were cellular process, single-organism process, biological process, and metabolic process. In the terms of cellular component, the major categories included cell, cell part, and organelle. The most enriched categories in the terms of molecular function were binding, catalytic activity, and nucleic acid binding transcription factor activity. The top 20 enriched pathways according to KEGG results are shown in Figure 3E. We found that the most enriched pathways were the cancer-related pathways, PI3K-Akt signaling pathway, pathways related to HTLV-I infection, microRNA in cancer, and proteoglycans in cancer, as well as MAPK signaling pathway.

### MiRNA–mRNA Network and Validation of DE miRNAs Which Are Related to the PI3K-Akt Signaling Pathway

**FIGURE 4:**
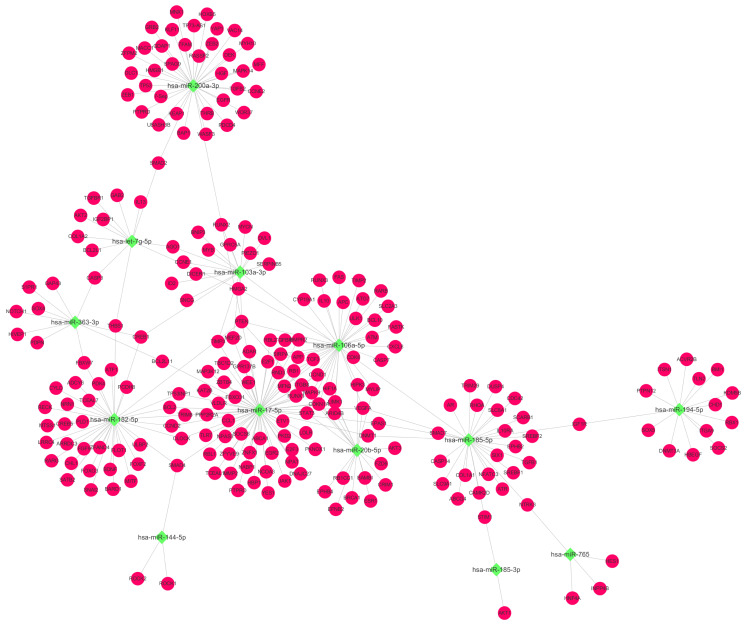
EXO-miRNA and mRNA interaction network analysis. The network targeting on miRNAs which were involved in the PI3K-Akt signaling pathway and their corresponding targets. Green squares and red round nodes indicate miRNAs and mRNAs, respectively.

**FIGURE 5:**
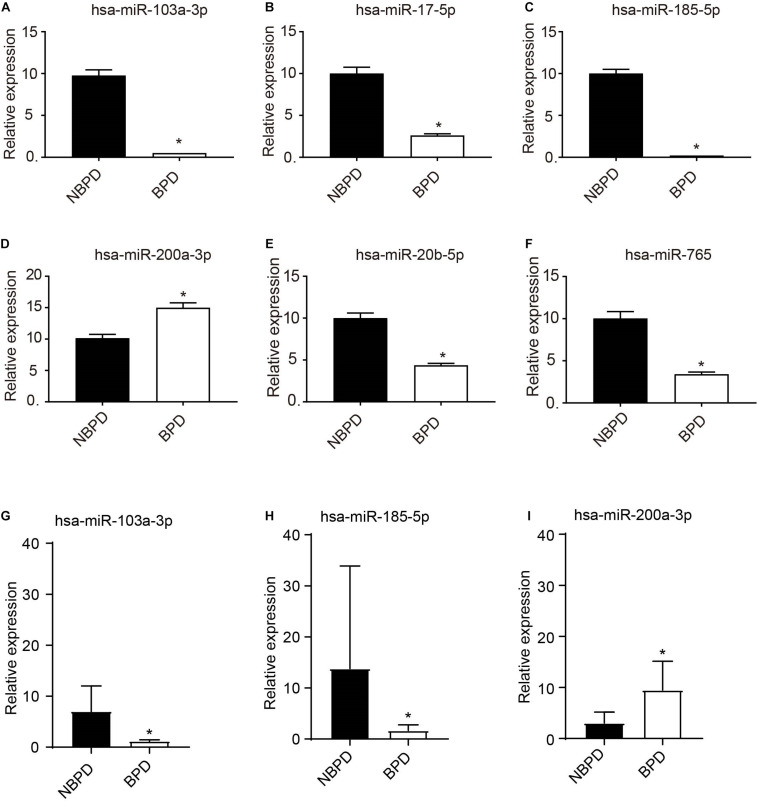
Validation of expression of EXO-miRNAs by RT-qPCR. **(A–F)** Expression of 6 EXO-miRNAs was determined in the exosomes which were used for sequencing. *n* = 4. **(G–I)** Expressions of hsa-miR-103a-3p, hsa-miR-185-5p, and hsa-miR-200a-3p were further validated in an additional non-sequencing EXO-miRNAs samples from NBPD and BPD infants. *n* = 8. *P < 00.05, BPD versus NBPD.

Based on the above KEGG results, we selected EXO-miRNAs related to both the PI3K-Akt signaling pathway and inflammation pathway to generate the miRNA–mRNA interaction network. As shown in Figure 4, 13 miRNAs were involved in these pathways and nine of these miRNAs were predicted to have more than three binding sites with their target genes. To validate DE miRNAs in the exosomes, six EXO-miRNAs were chosen, including hsa-miR-103a-3p, hsa-miR-17-5p, hsa-miR-185-5p, has-miR-200a-3p, hsa-miR-20b-5p, and hsa-miR-765 for qRT-PCR analysis. As shown in Figures 5A–F, the expression of hsa-miR-765 was decreased in exosomes from the BPD group when compared with the NBPD group, which was opposite to the NGS results. Other five miRNAs (hsa-miR-103a-3p, hsa-miR-17-5p, hsa-miR-185-5p, miR-200a-3p, and hsa-miR-20b-5p and) showed similar change trends as in NGS analysis. In addition, among the six validated miRNAs, hsa-miR-103a-3p, and hsa-miR-185-5p showed the most significant reduction, while hsa-miR-200a-3p was the only one showing the increase. We further selected these three miRNAs to validate their expression in exosomes isolated from additional samples from the NBPD and BPD groups which were not used for sequencing. We found that expressions of hsa-miR-103a-3p, hsa-miR-185-5p, and hsa-miR-200a-3p were also consistent with the NGS results (Figures 5G–I).

### Effects of PI3K-Akt Signaling Pathway-Related DE EXO-miRNAs on *in vitro* Angiogenesis

**FIGURE 6:**
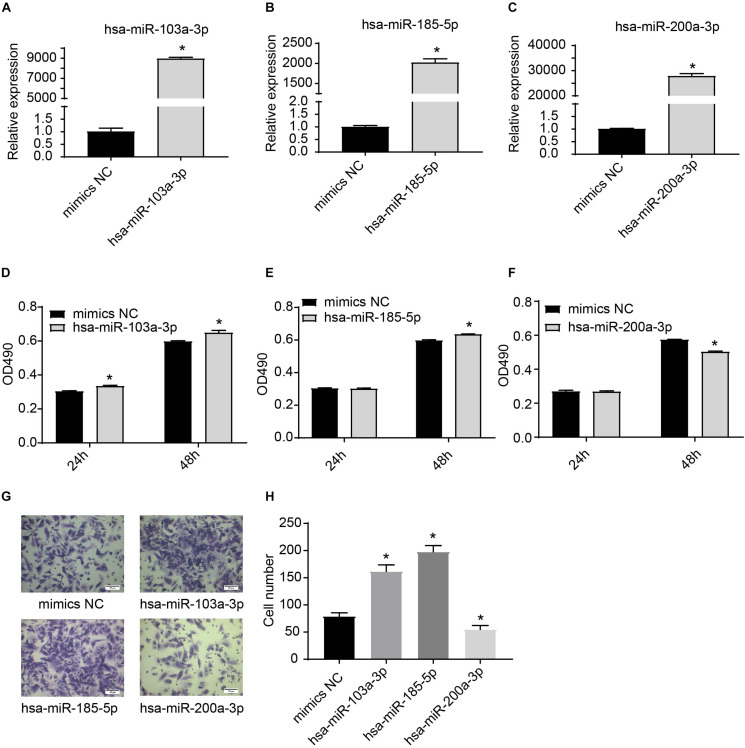
Effects of hsa-miR-103a-3p, hsa-miR-185-5p, and hsa-miR-200a-3p mimics on endothelial cell proliferation and migration. **(A–C)** Overexpression of hsa-miR-103a-3p, hsa-miR-185-5p, and hsa-miR-200a-3p in HUVECs. HUVECs were transfected with miRNA mimics for 24 h, and expression of miRNAs was determined by RT-qPCR. **(D–F)** Cell proliferation was determined at 24 h and 48 h after miRNA mimic transfection. Hsa-miR-103a-3p and hsa-miR-185-5p overexpression promoted endothelial cell proliferation, whereas hsa-miR-200a-3p overexpression suppressed endothelial cell proliferation. **(G,H)** Cell migration was determined at 6 h after miRNA mimic transfection. Hsa-miR-103a-3p and hsa-miR-185-5p overexpression promoted endothelial cell migration. Hsa-miR-200a-3p overexpression inhibited cell migration. *P < 0.05, versus mimics NC group. *n* = 3.

**FIGURE 7:**
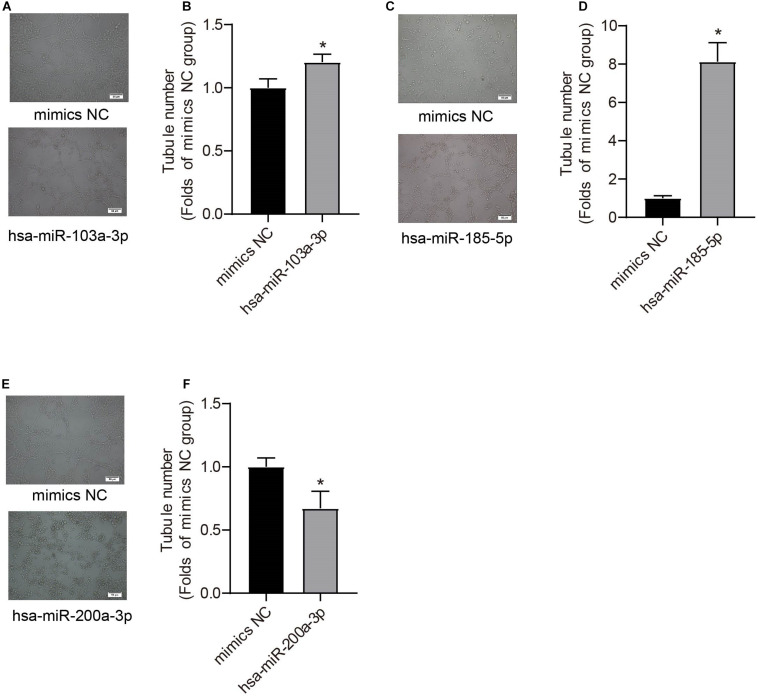
Effects of hsa-miR-103a-3p, hsa-miR-185-5p, and hsa-miR-200a-3p mimics on endothelial tube formation. Tube formation was determined at 48 h after miRNA mimic transfection. Hsa-miR-103a-3p **(A,B)** and hsa-miR-185-5p **(C,D)** overexpression increased endothelial tube formation, whereas hsa-miR-200a-3p overexpression **(E,F)** reduced tubule number. *P < 0.05, versus mimics NC group. *n* = 3.

We further used miRNA mimics to overexpress three EXO-miRNAs (has-sa-miR-103a-3p, hsa-miR-185-5p, and hsa-miR-200a-3p) to study their functions in endothelial cells (Figures 6A–C). We observed that transfection of three EXO-miRNA mimics elevated the levels of corresponding miRNAs (Figures 6A–C). Overexpression of hsa-miR-103a-3p and hsa-miR-185-5p significantly (*P* < 0.05) enhanced endothelial cell proliferation (Figures 6D,E). In contrast, overexpression of hsa-miR-200a-3p significantly (*P* < 0.05) inhibited endothelial cell proliferation (Figure 6F). Overexpression of hsa-miR-103a-3p and hsa-miR-185-5p dramatically (*P* < 0.05) promoted endothelial cell migration, whereas overexpression of hsa-miR-200a-3p significantly (*P* < 0.05) suppressed cell migration (Figures 6G,H). Similarly, overexpression of hsa-miR-103a-3p and hsa-miR-185-5p significantly (*P* < 0.05) enhanced tube formation, whereas overexpression of hsa-miR-200a-3p suppressed (*P* < 0.05) tube formation (Figure 7). These data uncovered the important function of EXO-miRNAs in regulation of endothelial cell function and angiogenesis.

### Hsa-miR-185-5p Negatively Regulated CDK6 mRNA Expression in HUVECs

**FIGURE 8:**
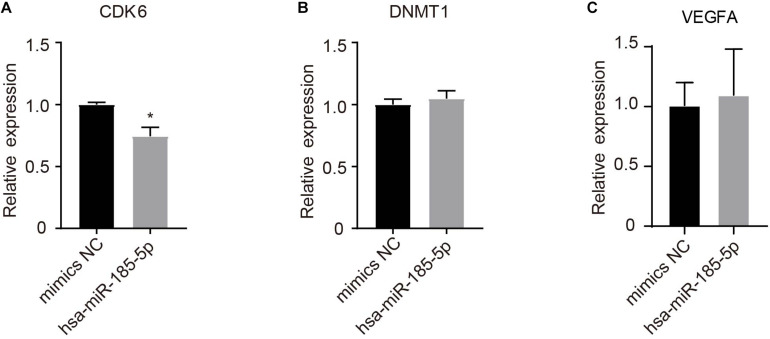
Determination of potential target genes of hsa-185-5p in HUVECs. Cells were transfected with hsa-185-5p mimics or mimics negative control for 24 h. CDK6 **(A)**, DNMT1 **(B)**, and VEGFA **(C)** mRNA levels were determined using qRT-PCR. *n* = 3, * versus mimics NC, *P* < 0.05.

A total of 39 target genes were predicted for hsa-miR-185-5p in HUVECs (Supplementary Table 5). As shown in Figure 8, hsa-miR-185-5p mimics significantly (*P* < 0.05) reduced CDK6, but not DNMT1 and VEGFA mRNA levels as compared with mimics negative control (mimics NC).

## Discussion

To the best of our knowledge, this is the first report demonstrating the roles of the DE EXO-miRNAs in umbilical cord blood from VPI with BPD. We found that exosomes isolated from umbilical cord blood of the BPD group significantly impaired endothelial angiogenesis when compared with the NBPD group. We also identified a set of DE EXO-miRNA in the BPD group with 90 downregulated (e.g., hsa-miR-103a-3p and hsa-miR-185-5p) and 328 upregulated (e.g., hsa-miR-200a-3p) miRNAs in BPD infants. These DE EXO-miRNAs are highly related to the PI3K-Akt signaling pathway. We further observed that overexpression of hsa-miR-103a-3p and hsa-miR-185-5p enhanced the proliferation, migration, and tube formation of endothelial cells, whereas overexpression of hsa-miR-200a-3p inhibited these angiogenic responses. We also identified CDK6 as a target gene of hsa-miR-185-5p. These data support that exosomes from VPI with BPD impair angiogenesis via EXO-miRNAs, which might lead to adverse outcomes of VPI with BPD. Future investigations are urgent to elucidate the potential of these DE EXO-miRNAs as the predictive biomarkers and therapeutic targets for VPI with BPD.

Therapeutic approaches for respiratory care, such as application of antenatal steroids and surfactant, continuous positive airway pressure therapy, and advanced ventilator device, have greatly improved the survival of preterm infants (Matthay and Abman, 2018). However, the increased survival of preterm infants potently increases the incidence of BPD (Stoll et al., 2015). Thus, there is an urgent need to find the biomarkers and effective therapeutic strategies for BPD. Exosome-based therapy has emerged as a potential tool for neonatal lung injury, including BPD (Willis et al., 2018). This is supported by the observation that exosomes derived from mesenchymal stem cells (MSC) protect against experimental BPD in mice (Panfoli et al., 2016), while exosomes derived from endothelial progenitor cells improve angiogenic activity of pulmonary microvascular endothelial cells in the *in vitro* model of BPD induced by hyperoxia (Zhang et al., 2019). Umbilical cord blood is an important source of these stem cells and exosomes. Exosomes isolated from umbilical cord blood can promote angiogenesis and wound healing (Hu et al., 2018), implying that exosomes derived from umbilical cord blood might participate in lung development. However, the exact roles of exosomes derived from preterm infants’ umbilical cord blood in the development of BPD remains elusive.

Bronchopulmonary dysplasia changes the contents and size of exosomes in the body fluids of infants as compared with the full-term control (Lal et al., 2018). In contrast, our results showed that the exosomes’ size between two groups is comparable. We found that exosomes derived from umbilical cord blood in the NBPD group promoted endothelial angiogenic responses, indicating that umbilical cord blood-derived exosomes from NBPD contains pro-angiogenic factors similar to those in the full-term infants (Hu et al., 2018). Our observation that exosomes from the BPD group impaired endothelial angiogenic responses suggests that the contents of bioactive molecules in exosomes of BPD are detrimental to the fetal vascular function. This is supported by a recent report by Genschmer et al. (2019) who showed that exosomes from the tracheal aspirate of intubated neonates with severe BPD could transfer mice into the BPD phenotype. Thus, the contents of exosomes could be the potential therapeutic targets and biomarkers of BPD development.

EXO-miRNA expression has been profiled in the body fluids of both human infants and animals with BPD (Bhaskaran et al., 2012; Braun et al., 2018). Developing a non-invasive procedure is ethically critical to obtaining clinical samples to investigate the role of miRNAs in the BPD development. This study demonstrates, for the first time, that collecting EXO-miRNA from umbilical cord blood from the very preterm infants with BPD and without BPD is a non-invasive and safe approach.

We have identified a set of DE EXO-miRNAs. These DE miRNAs identified in our study are distinct from those previously reported in the bronchoalveolar lavage fluid of infants with and without BPD (Lal et al., 2018). The discrepancy may be due to different cell and tissue origins of miRNAs (Landskroner-Eiger et al., 2013).

The DE EXO-miRNAs identified in the present study were predicted to potentially regulate a variety of signaling pathways, including PI3K-Akt signaling pathway, HTLV-I infection, and MAPK signaling pathway. Among these pathways, PI3K-Akt, inflammatory, and MAPK signaling pathways are closely related to angiogenesis (Sun et al., 2018). Among DE EXO-miRNAs that participated in these angiogenesis-relevant pathways, our data revealed that hsa-miR-103a-3p and hsa-miR-185-5p decreased most significantly and hsa-miR-200a-3p was the only miRNA which showed an increase in expression in the BPD samples. In addition, this study also showed that both hsa-miR-103a-3p and hsa-miR-185-5p overexpression significantly promoted endothelial cell proliferation, migration, and tube formation, but overexpression of hsa-miR-200a-3p exerted opposite effects on these angiogenesis indexes. Our results indicate that umbilical cord blood-derived exosomes from VPI with BPD impaired angiogenesis, which was potentially through dysregulation of hsa-miR-103a-3p, hsa-miR-185-5p, and hsa-miR-200a-3p.

Overexpression of hsa-miR-103a-3p is associated with the metastasis of breast cancer (Chang et al., 2016) and many other types of cancer (Liang et al., 2015; Fu et al., 2020). Hsa-miR-103a-3p expression is negatively regulated by inflammation (Cheng and Wang, 2020). As inflammation is a major cause of the pathogenesis of BPD, it is possible that inflammation in BPD decreased EXO-hsa-miR-103a-3p in umbilical cord blood from BPD infants. Given that hsa-miR-103a-3p responded to hypoxia and targeted argonaute 1 (AGO1) to promote angiogenesis (Chen et al., 2013), and EXO-miR-103a increases angiogenesis in gastric cancer targeting c-MYB (Liang et al., 2015), downregulation of exosomal hsa-miR-103a-3p in the BPD group is likely to suppress fetal angiogenesis. Our finding that hsa-miR-185-5p overexpression promotes angiogenesis is inconsistent with the previous report, which demonstrates that miR-185-5p suppresses the expression of vascular endothelial growth factor A (VEGFA), inhibiting angiogenesis in human ovarian microvascular endothelial cells (Wei and Zhao, 2020). In our study, hsa-miR-185-5p overexpression did not significantly change VEGFA mRNA expression in HUVECs. One explanation for this discrepancy is that the different endothelial cell types (human ovarian microvascular endothelial cells vs. HUVECs) were used. We identified CDK6 mRNA as a target of hsa-miR-185-5p. CDK6 can either promote or inhibit cell proliferation depending on its interaction with CDK4/6 inhibitor p16^ink4av^ (Kollmann et al., 2013). The roles of hsa-miR-185-5p/CDK6 signaling in angiogenesis have yet to be dissected. Consistent with our results, Chang et al. also found that overexpression of miR-200a-3p impaired angiogenesis (Chang et al., 2020). Our results show that overexpression of hsa-miR-103a-3p and hsa-miR-185-5p might rescue PBD-impaired fetal angiogenesis. However, further investigation is needed to reveal the role of their target genes in regulating angiogenesis in PBD.

## Data Availability Statement

The datasets presented in this study can be found in online repositories. The names of the repository/repositories and accession number(s) can be found below: NCBI GEO, accession number GSE166762.

## Ethics Statement

The studies involving human participants were reviewed and approved by the Ethics Review Board of the Third Affiliated Hospital of Guangzhou Medical University. Written informed consent to participate in this study was provided by the participants’ legal guardian/next of kin.

## Author Contributions

X-QZ, JZ, and Q-LC conceived this study and designed the experiments. X-QZ wrote the manuscript. JZ, Z-GC, and Q-LC revised the manuscript. X-QZ, Z-GC, and QY performed the experiments. C-HJ, Z-YL, and JG collected the samples and recorded clinical data. X-HL, H-LW, and C-YL analyzed the data and revised the manuscript. All authors contributed to the article and approved the submitted version.

## Conflict of Interest

The authors declare that the research was conducted in the absence of any commercial or financial relationships that could be construed as a potential conflict of interest.

## Footnotes

1 http://mirtarbase.cuhk.edu.cn/php/index.php
