# Supplementary material for: Umbilical Cord Blood-Derived Exosomes From Very Preterm Infants With Bronchopulmonary Dysplasia Impaired Endothelial Angiogenesis: Roles of Exosomal MicroRNAs
Source: Front Cell Dev Biol. 2021 Mar 25;9:637248. doi: 10.3389/fcell.2021.637248 (PMC8027316; doi:10.3389/fcell.2021.637248)
Supplement: Supplementary file 1 [file Table_1.DOCX]

Table S1. Primer sequences for EXO-miRNAs target genes determination.

| Primers | Sequences (5'-> 3') |
| --- | --- |
| CDK6 | Forward: TGCACAGTGTCACGAACAGA |
|  | Reverse: ACCTCGGAGAAGCTGAAACA |
| DNMT1 | Forward: GTGGGGGACTGTGTCTCTGT |
|  | Reverse: TGAAAGCTGCATGTCCTCAC |
| VEGFA | Forward: TTGCCTTGCTGCTCTACCTCCA |
|  | Reverse: GATGGCAGTAGCTGCGCTGATA |
