# Supplementary material for: Umbilical Cord Blood-Derived Exosomes From Very Preterm Infants With Bronchopulmonary Dysplasia Impaired Endothelial Angiogenesis: Roles of Exosomal MicroRNAs
Source: Front Cell Dev Biol. 2021 Mar 25;9:637248. doi: 10.3389/fcell.2021.637248 (PMC8027316; doi:10.3389/fcell.2021.637248)
Supplement: Supplementary file 2 [file Table_2.DOCX]

Table S2. Summary of sequencing.

| ﻿Sample | Raw Reads | Clean reads | Raw bases (G) | Clean bases (G) | Error (%) | Q20 (%) | Q30 (%) | GC (%) |
| --- | --- | --- | --- | --- | --- | --- | --- | --- |
| NBPD | 20,995,356 | 20,912,199 | 1.049 | 1.046 | 0.01 | 97.78 | 95.77 | 51.89 |
| BPD | 20,656,011 | 20,435,555 | 1.033 | 1.022 | 0.01 | 97.34 | 94.77 | 49.74 |
